# Supplementary material for: An Electrochemical Aptasensor Integrating Zeolitic Imidazolate Framework for Highly Selective Detection of Bioaerosols
Source: Biosensors (Basel). 2022 Sep 4;12(9):725. doi: 10.3390/bios12090725 (PMC9496278; doi:10.3390/bios12090725)
Supplement: Supplementary file 1 [file biosensors-12-00725-s001.zip › biosensors-1877236-supplementary.pdf]

Supporting Information

Section 1: Supplementary Figures

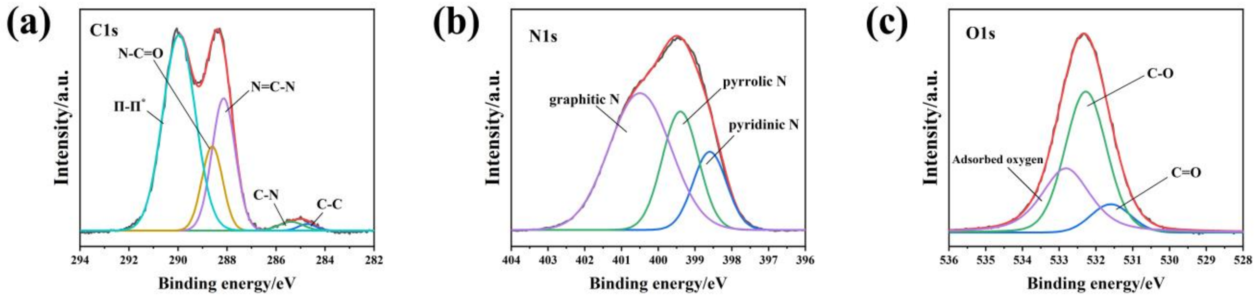

Figure S1. C1s, N1s and O1s XPS spectra of MCA.

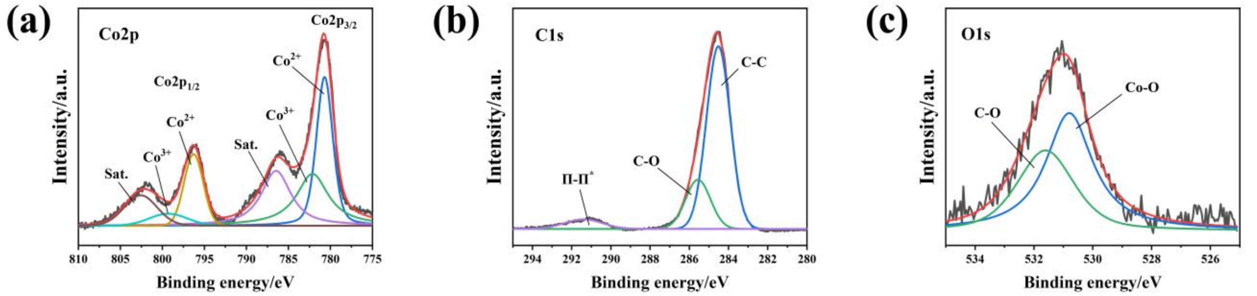

Figure S2. Co2p, C1s and O1s XPS spectra of ZIF-67.

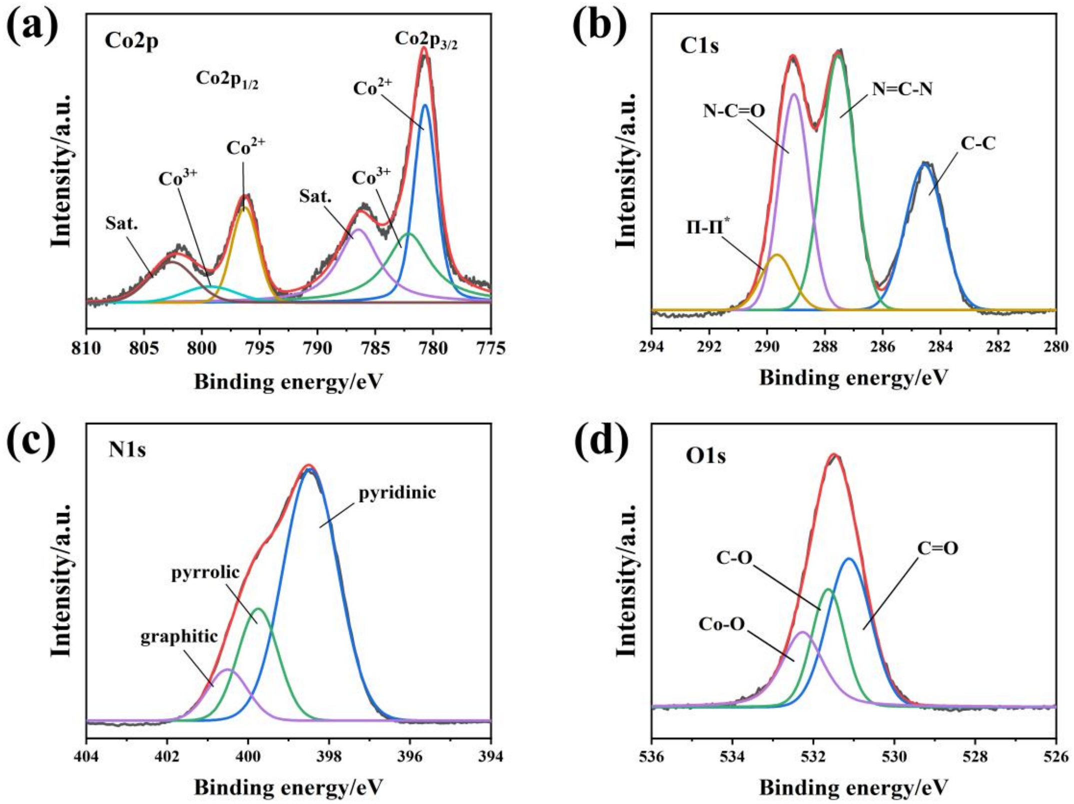

Figure S3. Co2p, C1s, N1s and O1s XPS spectra of ZM-2.

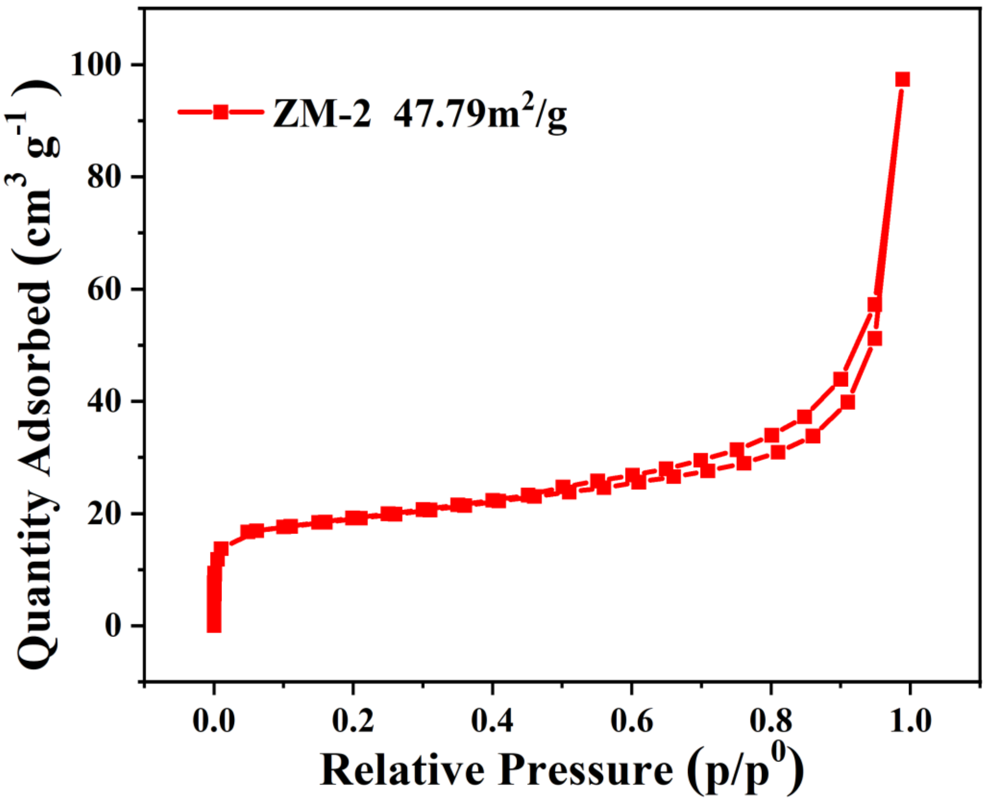

Figure S4. Nitrogen adsorption-desorption isotherms of ZM-2.

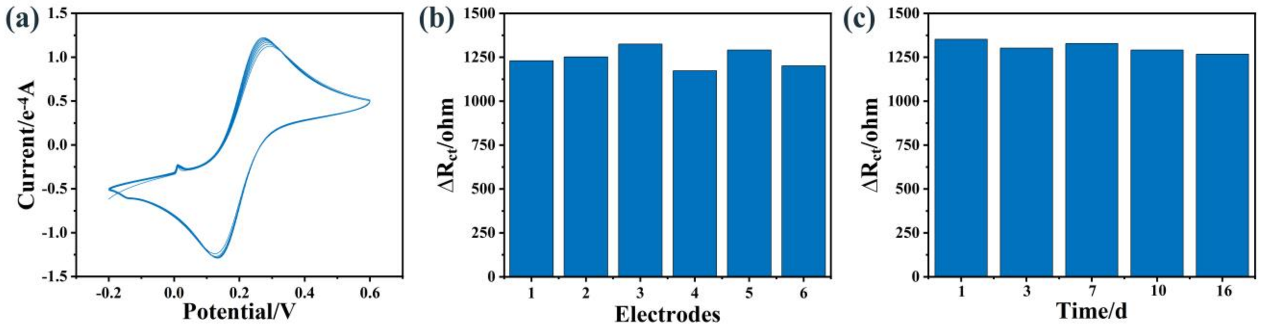

Figure S5. (a) The repeatability, (b) reproducibility, and (c) stability of the ZM-2-based aptasensor for detection of 100 nM ATP.

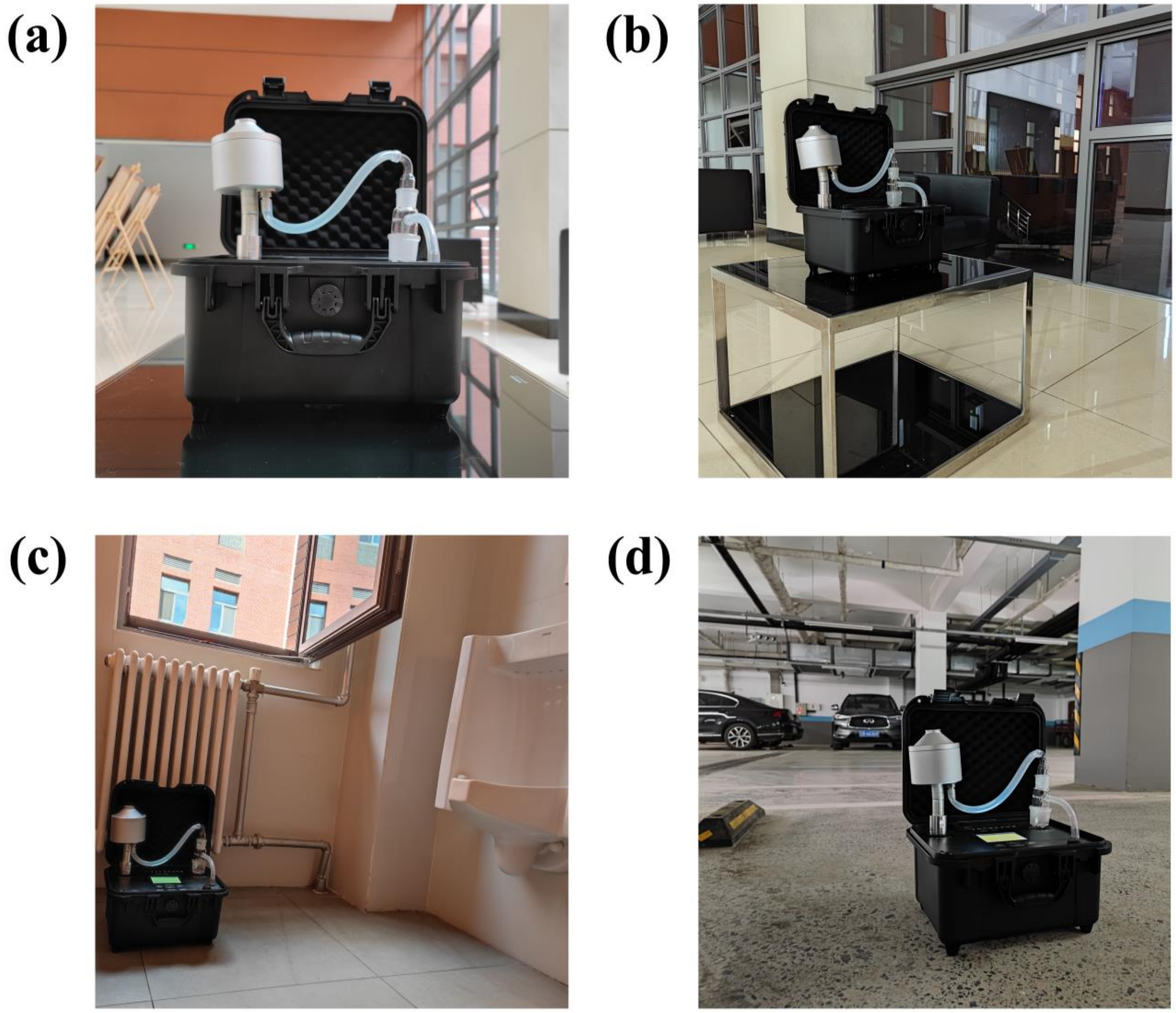

Figure S6. Digital images of bioaerosol collecting from different places or time. (a) Hall (day); (b) Hall (night); (c) Toilet; (d) Garage.

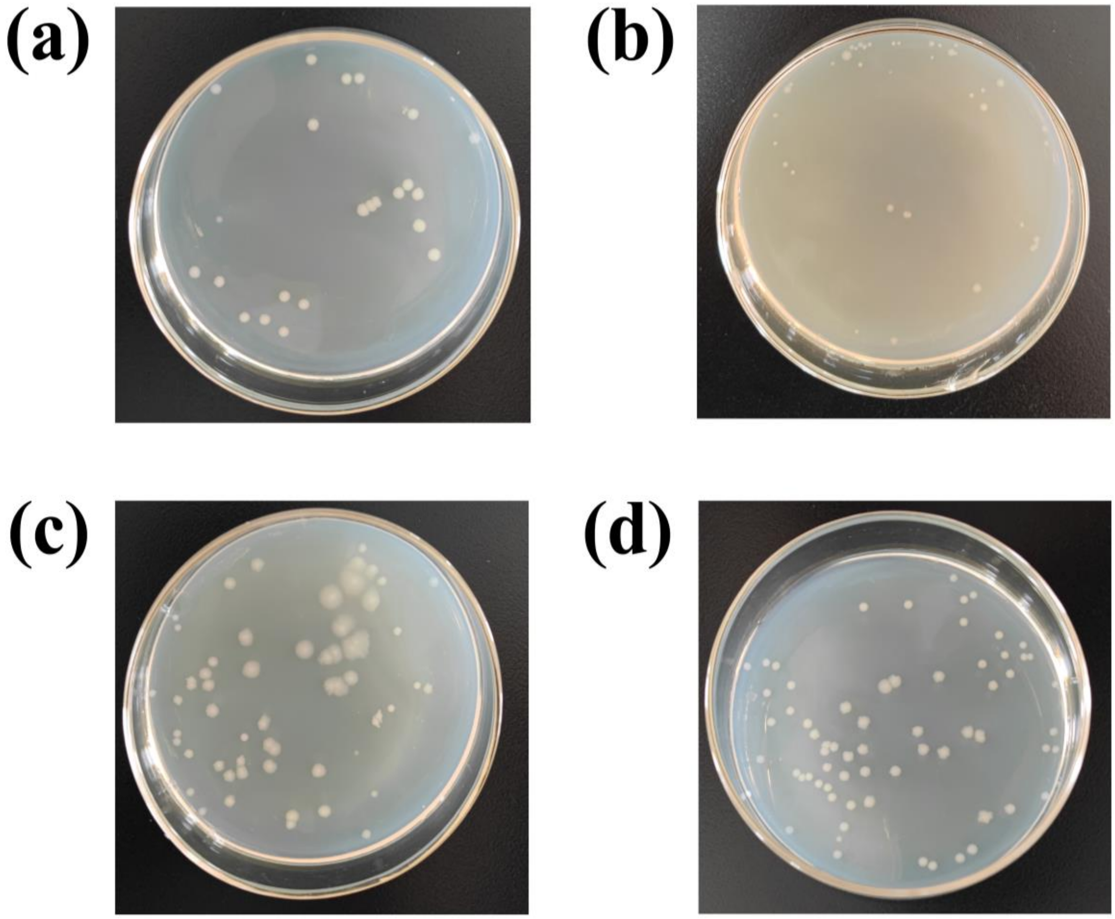

Figure S7. Digital images of bacterial bioaerosol culture plates collected from different places or time. (a) Hall (day); (b) Hall (night); (c) Toilet; (d) Garage.

Section 2: Supplementary Table

Supplementary Table S1. Summary of the oligonucleotides used for aptasensor design.

DNA oligonucleotide	Sequence (5′-3′)	Note
Apt	ACCTGGGGGAGT<br>ATTGCGGAGGAAGGT	Original sequence of ATP DNA aptamer
FApt	ACCTGGGGGAGTATTGCGGAGGAAGGT-FAM	FAM tagged part of ATP DNA aptamer
MApt	ACCTAAAAAAGTATTGCAAAAAAAGGT	Mutation sequence of ATP DNA aptamer

Supplementary Table S2. Various detection methods for ATP in some reported works.

Detection method	Detection range	LOD	Ref
EC/MOF	100 nm–1000 μM	3.9 nM	[37]
RLS/aptasensor	2.5–75 nM	0.046 nM	[38]
ECL/QDs	8–2000 nM	7.6 nM	[39]
CD/aptasensor	1.5–4.2 mM	0.2 mM	[40]
FL/GO	0.5–250 μM	100 nM	[41]
EC/exonuclease	0.1–20 nM	34 pM	[42]
ZIF-67-MCA/aptasensor	0.1–100 nM	0.11 nM	this work
